# Driving anger and its relationships with type A behavior patterns and trait anger: Differences between professional and non-professional drivers

**DOI:** 10.1371/journal.pone.0189793

**Published:** 2017-12-18

**Authors:** Zhongxiang Feng, Miaomiao Yang, Changxi Ma, Kang Jiang, Yewei Lei, Wenjuan Huang, Zhipeng Huang, Jingjing Zhan, Muxiong Zhou

**Affiliations:** 1 School of Automobile and Traffic Engineering, Hefei University of Technology, Hefei, Anhui, PR China; 2 School of Traffic and Transportation, Lanzhou Jiaotong University, Lanzhou, Gansu, PR China; 3 Traffic Management Research Institute of the Ministry of Public Security, Wuxi, Jiangsu, PR China; Beihang University, CHINA

## Abstract

The present study examined the types of situations that caused Chinese professional and non-professional drivers to become angry and investigated the differences in driving-elicited anger, considering the influences of type A behavior pattern and trait anger between the two groups. The 20-item revised Driving Anger Scale (DAS) was used to assess a sample of 232 drivers (57% professional, 43% non-professional). The non-professional drivers reported significantly higher levels of anger than the professional drivers on the overall Driving Anger Scale (DAS) and the traffic obstructions and discourtesy subscales. In both groups, the preferred driving speeds were positively related to driving anger. Furthermore, drivers with a type A personality exhibited higher overall driving anger scores and higher anger scores in response to traffic obstructions and slow driving than drivers with a type B personality. Trait anger was significantly related to driving anger in both groups. In the non-professional group, type A behavior patterns (TABPs) and time hurry (TH) were positively correlated with anger evoked by slow driving. In the professional group, TABPs, TH and competitive hostility (CH) were positively related to driving anger, and the TABPs exerted an indirect effect on driving anger by mediating the influence of trait anger. Overall, these findings provide a theoretical basis for implementing targeted interventions for driving anger in both professional and non-professional drivers.

## Introduction

Due to the rapid development of the economy and the increasing number of cars, travelling has become more convenient. However, there are numerous traffic accidents. Human factors play an important role in 92–94% of road traffic accidents [1]. Some studies have shown that the drivers’ psychological and physiological conditions contribute to their driving behaviors [2,3]. In particular, psychological conditions, such as the drivers’ characteristics, personalities, personal habits, and temporary emotions, and pressure and fatigue induced by the external environment significantly influence driving behaviors. Due to the increasingly rapid pace of modern urban life, people accumulate stress and negative emotions from their daily lives or work. Road rage, which is a negative emotion that occurs while driving, has become a common occurrence worldwide [4]. Since the mid-1990s, this phenomenon has begun to draw media attention. For example, in 1994, psychologists from Colorado State University defined driving anger as the propensity to become angry while driving [5]. However, driving anger is likely more common than currently believed and is likely experienced regularly by normal drivers. A previous study found that 89% of 270 drivers admitted to occasionally committing aggressive violations, such as chasing other cars, displaying hostility toward other drivers or sounding the horn to indicate their annoyance with the other drivers [6]. Similarly, a study conducted with a diary approach to record driving anger [7] indicated that 85% of 100 drivers experienced anger while driving over a 2-week period. Furthermore a strong link has been found between anger while driving and subsequent near accidents. Additional studies have indicated that driving anger could easily cause drivers to engage in aggressive and risky driving behaviors, such as speeding, tailgating, and the forcible overtaking of another vehicle [8–10]. Based on these studies, anger while driving likely interferes with the drivers’ attention, perception, information processing and driving performance; driving anger is correlated with crash-related conditions, such as the loss of concentration, loss of vehicular control, and near accidents, and directly or indirectly increases the likelihood of risky behaviors and accidents and, accordingly, affects road traffic safety [5,6,9].

Previously, Deffenbacher et al. established traffic-related scenarios to induce driving anger and developed the Driving Anger Scale (DAS), which is an effective measurement tool using cluster analysis that has established the foundation for subsequent studies [5]. Since the development of the original DAS, Britain, New Zealand, Spain and other countries have revised partial items and factor structures of the Driving Anger Scale (DAS) based on national and cultural differences and used the revised scales to conduct subsequent related studies [11–16]. In China, a study identified differences in the perception of driving anger between Chinese and German drivers. However, the German drivers exhibited a similar perception of driving anger to that of Westerners [17]. The inconsistent findings in these studies raised doubts as to whether all 33 of the original situations or factor structures in the DAS could adequately capture driving anger in Chinese drivers. In China, urban traffic is characterized by a mixture of motor vehicles, non-motor vehicles and pedestrians on the road. Thus, traffic scenarios that are consistent with the actual traffic characteristics in China may be more appropriate for revealing driving anger in Chinese drivers.

Driving anger is conceptualized as a personality trait that is correlated with trait anger [18]. In Japan, high levels of work stress arising from an effort-reward imbalance at work exert an indirect effect on driving anger by mediating the influence of trait anger [19]. In Malaysia, total trait anger scores were positively correlated with each driving anger subscale [20]. Trait anger, which has stable and enduring characteristics and occasionally plays a mediating role, was directly correlated with driving anger.

Researchers have associated personality types with traffic safety and driving behaviors. Drivers with a type A personality have received increasing attention [21]. Friedman and Rosenman [22], who are American cardiologists, were the first to identify the type A personality in a patient with coronary artery disease. The typical behavioral characteristics of a type A personality include intense ambition, aggressiveness, a competitive drive, a constant preoccupation with occupational deadlines, and a sense of time hurry (TH). The relative absence of these characteristics is defined as a type B personality behavioral pattern. Drivers with a type A personality tend to be impatient, are involved in more accidents, and receive more ticket [23]. Type A drivers were involved in more accidents and aggressive behaviors while driving on the road than type B drivers [24,25]. Furthermore, bus drivers with a type A personality performed more errors and violations while driving than those with a type B personality [26]. A type A personality is typically an aggressive personality that is formed by the interaction between the personality traits and the environment and has a negative effect on traffic safety. Although most studies exploring type A personality in drivers have focused on its correlation with accidents, to date, no studies have investigated its effect on driving anger. Thus, it is necessary to determine the effects of type A behavior patterns (TABPs) on anger behind the wheel.

Most researchers exploring the influences of demographic variables on driving anger have focused on gender or age differences. However, there exists a difference between professional and non-professional drivers. Naturally, this difference has drawn the attention of other researchers examining non-driving anger and has been shown to be significantly important. For example, differences in safety attitudes, behaviors, anxiety and perceived control were observed between professional and non-professional drivers [27]. In Israel, male taxi drivers and non-professional male drivers had different attitudes towards traffic violation penalties [28]. Electroencephalogram (EEG) changes during fatigue differed between professional and non-professional drivers [29]. Professional and non-professional drivers differ significantly in their skills, training, driving experiences, etc. Compared with non-professional drivers, professional drivers have higher workloads and conduct most of their driving within tight time schedules [30]. Furthermore, professional drivers have more exposure to higher traffic volumes on the road and receive more frequent group trainings. However, non-professionals drivers may be freer to choose their mode of driving and their target speed while driving [27]. Because driving anger in both groups has a great effect on road safety, it is reasonable to explore the differences in driving anger between professional and non-professional drivers. In addition, there are no differences in the educational and intervention methods used to address driving anger between these two groups. In particular, the traffic scenarios that evoke driving anger differently between professional and non-professional drivers in China are unknown. Furthermore, it is unclear whether there is a significant difference in driving anger between the two groups. An in-depth exploration is needed to determine the best approach to apply practical driving safety education. Therefore, investigating the levels of driving anger and the differences between these two groups has strong substantial impacts on driver selection and driver education.

Overall, the present study was undertaken to investigate the difference in the levels of driving anger between professional and non-professional drivers in China and to examine whether the differences in driving anger between the two groups are influenced by personality traits, namely, type A behavior pattern and trait anger. Finally, this study aimed to examine whether trait anger is a mediator that influences the relationship between driving anger and type A behavior patterns. In light of this, we hypothesize that;

H1. There is a significant difference in the driving anger experienced by professional and non-professional drivers, and professional drivers experience less anger overall.
H2. Trait anger and type A behavior patterns are significantly related to driving anger in both groups.
H3. TABPs exert an indirect effect on driving anger by mediating the influence of trait anger.

## Methods

### Participants and procedures

Data from non-professional drivers were collected by randomly distributing 200 questionnaires in gas stations, parking lots, large commercial centers, etc. in Hefei, Anhui Province; in addition, 150 professional drivers (all males) were randomly selected from the Hefei Passenger Transportation Co. Ltd. and asked to complete the questionnaires. The professional drivers were inter-city bus drivers, some of which drove medium distances between cities within Anhui Province and some of which drove long-distances on inter-province routes. All drivers drove at least 150 km one-way while on duty. The participants were first contacted by a researcher who explained the purpose of the study, the participant requirements, and the compensation. With the permission of the drivers, the interviewers distributed the questionnaires to the participants. The questionnaires were in the form of a self-report; thus, the participants were required to complete the questionnaire by following the instructions. Drivers were invited to participate in the survey with the assurance of anonymity and confidentiality, and the drivers were paid 30 yuan as a reward upon completing the questionnaire. Questionnaires with incorrect responses, lack of authenticity, or missing pages were considered invalid. Participants completed the questionnaires immediately upon receipt to ensure the authenticity of the responses.

In total, approximately 126 (63% of all questionnaires) completed questionnaires were returned from the non-professional drivers, and 150 (100%) completed questionnaires were returned from the professional drivers. In total, 232 questionnaires were valid (almost all males). The study included 132 professional drivers (57%) and 100 non-professional drivers (43%). The participants had an average age of 38.72 years (SD = 8.37) with an age range from 21 to 57. All participants had their driving licenses for more than one year (average = 12.96±9.06 yr).

### Materials

*The revised Driving Anger Scale*: The revised version of the DAS contained the most representative items from each subscale of the original 33-item DAS [5]. The translation of the original scale was conducted by three native Chinese speakers who were from the English department, and these translations were then back translated into English by a native English speaker. No major issues were identified during the translation process. Then, several representative traffic scenarios that induced driving anger in Chinese drivers, which were identified by interviewing 20 drivers, were added to the revised DAS. This process resulted in the addition of six new items, specifically, “someone changes lanes without turning on signal lights”, “someone changes lanes while crossing the solid white line at an intersection”, “someone parks park his car illegally on the road”, “non-motor vehicles occupy lanes designed for motor vehicles”, “someone parks park his car illegally at an intersection entrance or exit”, and “novice drivers drive too slowly on the road”. Then, a pre-test was carried out on 10 experienced drivers to identify ambiguous questions and questions that were irrelevant to the Chinese driving experience. The respondents were instructed to imagine the described traffic situation and assess their potential anger using a five-point Likert scale ranging from “1 = not at all” to “5 = very much”.

Overall, 39 items were selected based on this rationale (e.g., significance and suitability to the characteristics of the subscale concerned) and empirical criteria (e.g., discriminant coefficient or item-total correlation, reliability and validity test and exploratory factor analysis). A Principal Component Analysis (PCA) was performed to determine the factor structure, and all items were subjected to a factor analysis through varimax rotation.

Among the remaining items were ambiguous questions and questions that were irrelevant to the Chinese driving experience, namely, “someone makes an obscene gesture towards you”, “someone beeps at you” and “the police drive close by.” The “police presence” and “hostile gestures” factors from the original DAS were eliminated because anger induced by related items was low. Therefore, in addition to the those listed above, the items “a police officer pulls you over”, “a police driving close by” and “a police car is watching traffic from a hidden position” were eliminated. Thus, the revised version of the DAS consisted of 20 items that were grouped into the following four subscales: traffic obstructions, discourtesy, illegal driving and slow driving. After conducting the PCA, the Kaiser-Meyer-Olkin (KMO) measurement of sampling adequacy was 0.89, and the Bartlett Test of Sphericity was significant (*p*< 0.01). All factor loadings were greater than 0.45. The reliability analysis generated a Cronbach’s alpha coefficient of internal consistency of 0.90 for the total scale, and all subscales exhibited acceptable self-consistency, with Cronbach’s alpha values ranging from 0.75 to 0.81. The Cronbach’ alpha value was greater than 0.7 for the total scale and for each item, thus indicating an acceptable degree of reliability [31]. The correlations between each factor and the total scale reached statistical significance. An additional validity analysis of the revised 20-item scale was performed. The strength of correlations between the four factors ranged from low to low-medium (from 0.31 to 0.58), which indicated that the scale exhibited good differential validity. There was also a high degree of consistency between the content tested in the total scale and that for each factor (ranging from 0.64 to 0.84), which indicated that the revised scale had good criterion-related validity.

*Type A Behavior Pattern Scale*: The Chinese version of the Type A behavior pattern scale is a self-reported scale that was adapted from the Jenkins Security Survey [32] by Zhang [33]. TABPs have been characterized as hard-driving competitive behaviors, hostility, time urgency and a vigorous speech style. Under repeated testing, the correlation coefficient of the total scale score on the two tests was greater than 0.5, revealing a high test-retest reliability. The total questionnaire contains 60 items and the following three subscales: time hurry (TH), which includes 25 items and emphasizes TH; competition hostility (CH), which includes 25 items that highlight features, such as competitiveness, wariness and hostility; and a 10-item section for lie (L) detection. The respondents were asked to mark each item with a tick mark for “Agree” and a cross for “disagree” to determine which items fit the particular individual. First, the L scale score was calculated. If the score was 7 points or greater, the respondents were eliminated due to the lack of authenticity. The evaluation criteria for the types of personality depended on the total scores of TH and CH [26,33]. We define the types as follows: type A: 37 to 50 points; type A-: 30 to 36 points; mid-type: 27 to 29 points; type B-: 20 to 26 points; and type B: 1 to 19 points.

*Trait Anger Scale* (TAS): The TAS was selected as a measure of general and enduring feelings of anger. The Chinese version of the TAS was translated and revised from the original English version of the State-Trait Anger Expression Inventory-2 [34] by Tao [35]. According to the Cronbach’s alpha coefficient, the internal consistency of the total scale was 0.80. The TAS is a 10-item scale designed to measure people’s trait propensities for anger in daily life (for example, “I am a hot-headed person”). Ratings are made on a four-point Likert Scale ranging from “1 = almost never” to “4 = almost always”.

*Driving behavior*: The questionnaire also contained a survey regarding driving behaviors. The drivers were required to report their preferred driving speeds on three types of speed-limit roads (an expressway with a speed limit of 120 km/h, a highway with a speed limit of 80 km/h, and an urban road with a speed limit of 60 km/h).

*Demographic information*: Demographic details, including gender, age, professional or non-professional driving status and driver information, such as driving experience, mileage in the past year, etc., were obtained from all participants.

## Results

### Demographics and preferred driving speeds

Table 1 shows the basic demographics of the professional and non-professional drivers who participated in this study. The differences in the demographic composition of age, driving experience and miles driven over the previous year were investigated between the non-professional and professional driver groups. As illustrated in Table 1, there were differences in the age composition (*F* = 33.89, *p*<0.001), driving experience (*F* = 11.02, *p*<0.001), and mileage in the past year (*F* = 75.13, *p*<0.001) between the two driver groups. Professional drivers reported a higher average age, more driving experience and more mileage driven in the past year than the non-professional drivers, indicating that the professional drivers had a higher exposure to road traffic vehicles than the non-professional drivers, which is consistent with the results presented by Nordfjærn et al. [27].

**Table 1:** Demographics and preferred driving speeds of the two groups.

Variable	Mean (SD)		*t*-value
	Professional	Non-professional	
Age	42.94 (5.04)	33.15 (8.64)	10.10****
Driving experience	18.81 (6.61)	5.23 (5.26)	17.42****
Mileage in the past year	148681 (54,500)	12962 (14,916)	27.29****
Preferred speed on expressway (120 km∕h)	99.77 (8.32)	108.67 (11.13)	-6.69****
Preferred speed on highway (80 km∕h)	69.94 (8.15)	71.45 (9.05)	-1.33
Preferred speed on urban road (60 km∕h)	51.92 (9.77)	51.93 (8.08)	-0.005

* *p*<0.05

** *p*<0.01

*** *p*<0.005

**** *p*<0.001

Participants reported their preferred driving speeds on the following three different types of roads: expressways, highways and urban roads (Table 1). As expected, the average speed of expressway driving was the highest in both groups with an average of 108.67 km/h in the non-professional driver group and 99.77 km/h in the professional driver group, which was consistent with the speed limit of 120 km/h. Furthermore, an independent samples t-test (two-tailed) indicated that on expressways, the non-professional drivers preferred significant higher driving speeds than the professional drivers (*F* = 16.15, *p*<0.001). In contrast, both the non-professional and professional drivers preferred similar driving speeds on highways and urban roads.

### Driving anger and differences between the two groups

The means and standard deviations of the professional drivers and non-professional drivers’ scores on the revised 20-item DAS are presented in Table 2. Illegal driving was considered a factor that induces driving anger and had the highest score of the four driving anger subscales in the professional driver group with an average subscale score of 2.74 (SD = 0.96). Furthermore, in this subscale, the item “someone parks his car at an intersection entrance or exit” was rated 3.23 (SD = 1.48), which was the highest scoring item of all 20 items with respect to provoking professional drivers’ anger. Traffic obstructions were the second major factor to induce professional drivers’ anger, in which the item “the reminding flag is not set when the road is under reconstruction” was another high anger-inducing scenario with a rating of 3.23(SD = 1.35). The least anger-inducing situation for the professional drivers was slow driving, which had the lowest average anger ratings (M = 1.88; SD = 0.69) of the four subscales. However, for the non-professional drivers, traffic obstructions were the primary inducing factors of driving anger and had the highest score of the four factors with a subscale score of 2.87 (SD = 0.92). The item on the traffic obstruction subscale “you hit an unmarked deep pothole when driving” had a score of 3.39 (SD = 1.35), which was the highest scoring anger-inducing item among non-professional drivers. The slow driving subscale had the lowest average anger ratings in the group of non-professional drivers (M = 2.23; SD = 0.91), which was similar to the professional drivers. In both groups, driving anger was highly provoked by two items, namely, “the reminding flag is not set when the road is under reconstruction” and “someone parks his car at an intersection entrance or exit”, and both items had a score greater than 3.

**Table 2:** Differences in the DAS scores between the professional and non-professional driver groups.

Item	Professional N = 132, M (SD)	Non-professional N = 100, M (SD)	*F*-value
**Traffic obstructions (α = 0.79)**	2.67(0.84)	2.87(0.92)	4.47*
Other cars block traffic in the parking process.	2.00(1.17)	2.18(1.11)	
You hit an unmarked deep pothole when driving.	2.94(1.31)	3.39(1.35)	
You drive behind a badly smoking vehicle and the smoke blocks your sight.	2.48(1.27)	2.72(1.26)	
You drive behind a large truck which occupies the lane.	2.59(1.19)	2.83(1.30)	
The reminding flag is not set when the road is under reconstruction.	3.23(1.35)	3.11(1.30)	
A truck is kicking up gravel at your car.	2.77(1.33)	3.02(1.24)	
**Discourtesy(α = 0.80)**	1.94(0.88)	2.34(0.85)	4.98*
Someone cuts right in front of you on the freeway.	1.79(1.17)	1.99(1.23)	
Someone cuts in and takes the parking spot you have been waiting for.	2.30(1.31)	2.95(1.31)	
Someone tries to speed up to drive in front of you.	1.65(0.92)	1.71(0.92)	
Someone changes lanes in front of you when there is no one behind you.	2.08(1.09)	2.57(1.24)	
When you drive normally, someone honks at you.	1.89(1.12)	2.51(1.89)	
**Illegal driving(α = 0.81)**	2.74(0.94)	2.62(0.94)	1.93
Other vehicles run a red light.	3.18(1.55)	2.63(1.47)	
Someone parks his car in an intersection of entrance or exit.	3.23(1.48)	3.16(1.32)	
Someone changes lanes without turning on signal lights.	2.67(1.17)	2.97(1.25)	
Someone is speeding on a speed-limit road.	2.34(1.22)	2.07(1.23)	
Someone parks his car illegally on the road.	2.08(1.27)	2.55(1.31)	
Someone changes lanes while crossing the solid white line in an intersection. an intersection.	2.25(1.13)	2.36(1.36)	
**Slow driving(α = 0.75)**	1.88(0.69)	2.23(0.91)	2.41
Someone in front of you does not start moving when the light turns green. turns green.	1.74(0.68)	2.21(1.21)	
A pedestrian walks slowly across the middle of the street, forcing you to slow down.	1.56(0.80)	1.97(0.98)	
Someone is driving too slowly in the passing lane.	2.34(1.06)	2.50(1.17)	
**Total driving anger(α = 0.90)**	2.39(0.68)	2.57(0.73)	5.26*

* *p*<0.05

Overall, compared with findings in the USA, Turkey and Malaysia [5,36,37], all drivers in China, regardless of whether they were professional drivers or non-professional drivers, reported lower levels of anger than the drivers in these other countries on similar subscales. Furthermore, for each dimension, the levels of driving anger were less than 3 in the Chinese drivers, which was similar to the results reported by Li, Yao, Jiang and Li [38], who found that a Chinese sample reported significantly lower levels of anger than those from countries, such as the USA, Turkey, etc. In the USA and Malaysia samples, the discourtesy subscale produced the highest means. However, the subscale with the highest mean score in the non-professional drivers in China was the traffic obstructions subscale, whereas for the professional drivers, the highest mean score was reported on the illegal driving subscale.

Because age, driving experience and mileage in the past year could potentially affect the differences in the driving-related variables between the two groups, the influence of the demographic characteristics was adjusted for in the subsequent analyses. A multivariate analysis of covariance (MANCOVA) was performed to investigate the differences in driving anger evoked by the traffic scenarios between the two driver groups. The MANCOVA was used to examine whether the main effect of being a professional or non-professional driver maintained significance when the interactions between the group status and its demographics were taken into consideration. A Box’s M test was conducted to investigate the homogeneity of the variance in the covariance matrices and revealed that the variances were equal (*F* = 1.62, *p*>0.05). Furthermore, the outcome of the MANCOVA indicated that the covariates of age, driving experience and mileage in the past year were not significant.

The specific differences in driving anger between the professional and non-professional drivers are reported in Table 2. There were significant differences in the traffic obstructions subscale (*F* = 4.47, *p*<0.05), the discourtesy subscale (*F* = 4.98, *p*<0.05) and the total scale scores between the two groups (*F* = 5.26, *p*<0.05). Furthermore, the non-professional group reported significantly higher levels of driving anger than the professional group. These results provide support for the first hypothesis, that there is a significant difference in the driving anger experienced by professional and non-professional drivers, and professional drivers experience less anger overall.

### Relationships among driving anger, demographics and preferred speed

A Pearson’s correlational analysis was performed to investigate the correlations between the four subscales or the total scale of driving anger and the demographics, such as age, driving experience and mileage in the past year. In Table 3, the left column provides information regarding the professional drivers, and the right column provides information regarding the non-professional drivers. The professional drivers’ age, driving experience and mileage in the past year were weakly and positively correlated with the driving anger scores. The non-professional drivers’ age and mileage were weakly and negatively correlated with driving anger, while driving experience was weakly and positively correlated with driving anger.

**Table 3:** The correlation coefficients among driving anger, demographics and preferred speeds.

	Traffic obstructions		Discourtesy		Illegal driving		Slow driving		Total score	
	Pro	Non- pro	Pro	Non- pro	Pro	Non- pro	Pro	Non- pro	Pro	Non- pro
Age	0.02	-0.14	0.05	-0.08	0.09	0.01	0.03	-0.01	0.07	-0.07
Driving experience	0.04	0.09	0.09	0.08	0.09	0.12	-0.02	0.09	0.08	0.12
Mileage in the past year(km)	0.11	0.01	0.03	-0.16	0.14	-0.05	-0.01	-0.05	0.11	-0.06
Preferred speed on expressway	0.35**	0.21*	0.58**	0.07	0.18*	-0.01	0.47**	0.11	0.46**	0.12
Preferred speed on highway	0.21*	0.20*	0.27**	0.23*	0.03	0.11	0.27**	0.21*	0.22*	0.22*
Preferred speed on urban road	0.27**	0.09	0.27**	0.09	0.09	-0.01	0.28**	0.15	0.26**	0.08

* *p*<0.05

* * *p*<0.01

The relationships between driving anger and the drivers’ preferred driving speed on the three different road types are presented in Table 3. On expressways, the preferred driving speeds of professional drivers were significantly and positively correlated with all subscales and the overall driving anger scores. On highways and urban roads, the preferred speeds of the professional drivers were significantly and positively correlated with all subscales, except for the illegal driving subscale, and positively correlated with the overall driving anger scores. In the non-professional driver group, only the preferred driving speed on highways displayed a significant correlation with the DAS scores, except for the illegal driving subscale, while the other correlations were not significant. As expected, the non-professional and professional drivers who reported higher speed preferences on roads are more likely to exhibit higher levels of anger than the drivers who preferred slower speeds.

### Driving anger, TABP and trait anger

The 232 participants were divided into three categories, namely, type A, middle and type B, according to the evaluation criteria of the TABP. One-way analysis of variance (ANOVA) was conducted to investigate the difference in driving anger among the three personality types. The personality differences in overall anger (*F*(2, 229) = 4.36, *p*<0.05), anger evoked by traffic obstructions (*F*(2, 229) = 4.59, *p*<0.05) and anger evoked by slow driving (*F*(2, 229) = 7.56, *p*<0.005) were significant. Then, the Least Significant Difference (LSD) method of multiple comparisons was performed to conduct pair-wise comparisons, and the results are shown in Table 4. Additional post hoc tests indicated that the drivers with a type A personality reported significantly higher levels of driving anger than the drivers with a type B personality on the traffic obstructions subscale, slow driving subscale and the total scale. Interestingly, drivers with a type A personality reported significantly higher levels of driving anger than those with a middle personality only when confronted with slow driving. Overall, drivers with type A personalities experienced the highest levels of anger, followed by those with middle-type personalities. The drivers who experienced the lowest levels of driving anger were those with type B personalities.

**Table 4:** The results of the multiple comparison tests.

Driving anger	Type A N = 114, M	Middle N = 39, M	Type B N = 79, M	*F*
Traffic obstructions	2.92	2.75	2.53	4.59*
Discourtesy	2.21	2.14	1.96	1.84
Illegal driving	2.75	2.81	2.56	1.32
Slow driving	2.23	1.95	1.78	7.56***
Total scale	2.59	2.49	2.28	4.36*

* *p*<0.05

*** *p*<0.005

A Pearson correlation analysis was calculated to test the correlations among driving anger, type A behavior pattern and trait anger. In Table 5, the left columns provide information regarding the professional drivers, and the right columns provide information for the non-professional drivers. Moderate relationships were found in the professional drivers between the CH subscale and the DAS. However, TH was significantly and positively correlated with the total DAS and the traffic obstructions subscale, suggesting that professional drivers who had higher levels of TH were likely to become angered over traffic obstruction situations. The TABP was found to be significantly and positively correlated at a small to moderate level with each driving anger subscale, particularly the total DAS, *r* = 0.34, *p*<0.01. Additionally, the correlation between the TABP and trait anger was significant, *r* = 0.38, *p*<0.01. These correlations suggest that professional drivers with high levels of TABPs are not only more likely to experience anger but also more likely to experience a more profound feeling of anger both on the road and in everyday life. As expected, a significantly positive correlation was observed between trait anger and driving anger, *r* = 0.55, *p*<0.01, indicating that professional drivers who reported high trait anger tended to produce elevated levels of driving anger.

**Table 5:** The correlation coefficients of driving anger and personality traits.

	Traffic obstructions		Discourtesy		Illegal driving		Slow driving		Total score	
	Pro	Non- pro	Pro	Non- pro	Pro	Non- pro	Pro	Non- pro	Pro	Non- pro
TH	0.25**	0.15	0.16	0.07	0.13	0.09	0.16	0.23*	0.23**	0.15
CH	0.36**	0.04	0.19*	0.01	0.18*	0.01	0.29**	0.15	0.32**	0.05
TABP	0.37**	0.11	0.22*	0.04	0.20*	0.05	0.29**	0.21*	0.34**	0.11
TAS	0.47**	0.14	0.52**	0.21*	0.35**	0.07	0.43**	0.33**	0.55**	0.20*

* *p*<0.05

** *p*<0.01

In non-professional drivers, the TABP and TH subscale displayed significant positive correlations with driving anger evoked by slow driving, indicating that non-professional drivers who scored high on TH were angered, in general, over situations that forced them to drive slowly. Only the total DAS and the two subscales of discourtesy and slow driving were significantly and positively correlated with trait anger. In particular, there was no significant correlation between TABP and trait anger. Therefore, the non-professional group was not included in the regression analyses.

### Hierarchical multiple regression analyses

To determine whether TABPs, as an independent variable, exert an indirect effect on driving anger by mediating the influence of trait anger in professional drivers, Baron and Kenny’s [39] four steps were performed. First, driving anger was regressed onto TABP. As shown in Table 6, a significantly positive relationship was observed between TABPs and driving anger in Model 1, which satisfied step 1. Next, trait anger was regressed onto TABP, and Model 2 showed that TABPs remained a significant predictor, which satisfied step 2. Then, driving anger was regressed onto TABP and trait anger in Model 3. A significant and positive relationship was observed between driving anger and trait anger, which satisfied step 3. This finding support hypothesis 2, that trait anger and type A behavior pattern are significantly related to driving anger in both groups. In this final model, TABP was no longer significant, thus supporting step 4. Finally, the Sobel z-test was performed, and the test was significant, *z* = 6.09, *p*<0.001, which indicated that trait anger significantly carried the effect of TABPs on driving anger. Because the four steps and the Sobel z-test were significant and confirmed the requirements for mediation, trait anger fully mediated the relationship between TABPs and road anger. This result provides support for the third hypothesis, that TABPs exert an indirect effect on driving anger by mediating the influence of trait anger.

**Table 6:** Standardized regression coefficients (*β*) in the hierarchical multiple regression models.

	Model 1 Driving anger	Model 2 Trait anger	Model 3 Driving anger
TABP	0.273****	0.375****	0.094
Trait anger			0.493****
*R*	0.273	0.375	0.535
*R*^2^	0.074	0.141	0.286
	*F* (1, 130) = 10.46***	*F* (1, 127) = 20.76****	*F* (2,126) = 25.27****

*** *p*<0.005

* ** * *p*<0.001

## Discussion

This study investigated the problem of road rage in professional and non-professional drivers in the Chinese context of a mixed traffic mode and captured the traffic scenarios that evoked greater driving anger in the two groups of drivers. Furthermore, significant differences in driving anger were revealed between the two driver groups after considering the differences in demographics. Additionally, drivers with a type A personality exhibited the typical characteristics of aggressiveness and time urgency, which increased their propensity toward anger while driving. Furthermore, consistent with our hypothesis, trait anger played a mediating role in the relationship between type A behavior pattern and driving anger in the sample of professional drivers.

Demographic statistical analysis showed there was a significant difference in the average age between the two groups. In general, most professional drivers ranged from 30 to 50 years of age. In contrast, in the present sample, the non-professional drivers were younger. The professional drivers apparently had more driving experience and miles driven in the previous year, which resulted from the nature of their work as professional drivers. By examining the preferred driving speeds on three types of roads, it was found that on expressways, the preferred driving speed in the non-professional group was 9 km/h higher than that in of the professional group. As previously mentioned, this finding appears to be linked to a Chinese traffic rule in which the maximum speed limits on expressways for large and medium-sized buses are lower than those for cars, and additionally, professional drivers face tighter speed restrictions than non-professional drivers due to company regulations and potential sanctions for speeding imposed by their company. In contrast, non-professionals drivers may be freer to choose their modes of driving and their target speeds while driving, which is consistent with the results reported by Nordfjærn et al. [27]. As a result, the differences observed in the demographic statistical analysis may explain some of the differences in driving anger between the professional and non-professional drivers.

The professional drivers in this study reported the highest anger propensities on the illegal driving subscale, followed closely by the traffic obstructions subscale, which is consistent with the results reported by Feng et al [40]. For the non-professional drivers, the traffic obstructions subscale exhibited the highest mean. These findings, however, were not consistent with previous studies conducted in the USA [5], the UK [41], New Zealand [14], France [42], Turkey [37] and Malaysia [24] in which discourteous actions of other drivers were among the most anger-provoking situations. However, the results of the non-professional drivers were consistent with the findings reported by Li, Yao, Jiang and Li [38], who found that a Chinese sample reported the highest anger when facing traffic obstructions. In China, traffic obstructions caused by road reconstruction are commonplace in large cities due to urban redevelopment and the construction of subways lines. Thus, drivers in China are more sensitive to these phenomena and express annoyance at delays and broken schedules caused by traffic obstructions, which could account for these results. Interestingly, the least anger-provoking situation was slow driving in both the professional and non-professional driver groups, which differed from the results obtained in New Zealand and Spain in which police presence evoked the lowest level of self-reported anger [14]. These findings may imply that the two groups tend to provide a socially acceptable response rather than expressing their true opinions. Additionally, both Chinese professional and non-professional drivers reported lower levels of anger across all subscales and the total DAS than samples from the USA [5], Turkey [37] and Malaysia [24]. Culture may account for certain differences. In the traditional Chinese culture, extreme emotions are typically disapproved of because they are perceived as pathogenic behaviors that disrupt the body’s natural harmony [43–45]. Accordingly, the disapproval of extreme emotions may have a strong influence on the Chinese individuals’ control over their expressions of anger [45].

Another interesting and surprising inference from our results was the significant difference in driving anger between the two driver groups. Non-professional drivers reported higher levels of anger than professional drivers on almost all items on the total DAS. In particular, after considering the demographic differences between the two driver groups, the differences in anger caused by traffic obstructions and discourtesy were significant. A potential explanation is that the particularity of the driving profession requires professional drivers to receive more educational training and subjects them to stricter rules and constraints on the road. Thus, professional drivers experience less anger than non-professional drivers in most traffic scenarios. This result may be instructive for controlling driving anger and designing interventions for both professional and non-professional drivers. For example, as an intervention to improve emotional control, it can be stipulated that non-professional drivers receive regular emotional management training, like professional drivers. Driving anger has been shown to significantly decline with age, driving experience and mileage [46,24]. Unfortunately, contradictory findings existed regarding the impact of demographics on driving anger in the present study, and no significant correlations were revealed between the demographics, such as age, driving experience and mileage, and driving anger in both the professional and the non-professional groups. These results were similar to the findings in a Spanish sample investigated by Sullman et al. [15], who found that anger did not significantly decline with age and miles driven, and are consistent with the results reported by Li et al. [47], who found no significant correlations between anger and age or driving experience in a Chinese sample. One explanation is that regional differences existed in the samples from these countries, suggesting that drivers were influenced by culture, traffic patterns and driving habits. Additionally, in this study, the selected sample (the professional group and non-professional group) was narrow with respect to range of age, driving experience and mileage, which resulted in no significant correlations.

Another finding consistent with previous studies [14,15,20] was that drivers who reported higher speed preferences were more likely to become angered over slower drivers. In particular, professional drivers face tighter restrictions on speed than non-professional drivers due to company regulations that perceive driving fast as a risk factor that induces anger while driving. Studies have shown that by exploring the relationships between factors related to traffic incidents, traffic management departments can better pinpoint their weakness, and consequently develop more effective, targeted countermeasures [48]. Therefore, in future work, studies exploring interventions in professional drivers with respect to driving anger should focus on those drivers who prefer higher speeds. In addition, the relevant departments should increase penalties for speeding violations to curb speeding tendencies.

Approximately half of the drivers had type A personalities, which appears to be consistent with the results of the interactions of modern social environmental factors and stable personality traits. In addition, compared with drivers with a type B personality, drivers who had a type A personality reported higher levels of driving anger, particularly on the traffic obstructions subscale and the slow driving subscale. The typical behavioral characteristics of type A personality may account for these results. In particular, aggressiveness and a sense of time urgency are the main characteristics of a type A personality, and these characteristics play a key role in evoking driving anger when situations related to traffic obstructions and slow driving cause delays in their schedules. Thus, drivers with a type A personality should receive more attention, and based on previous studies [24,25], future studies should investigate whether type A individuals are involved in more traffic accidents as a result of increased anger and aggression while driving.

In this study, the correlations between trait anger and driving anger in both the professional and non-professional driver groups support the premise that drivers with a high proclivity toward anger are also likely to experience elevated levels of anger behind the wheel. Additionally, the finding that trait anger correlates positively with driving anger has been corroborated by previous studies, such as Deffenbacher et al. [5,49,50], Sullman et al. [24], McLinton and Dollard [19], etc. In addition, the higher the scores reported by the professional drivers on the TABP and the competition hostility subscale, the higher their scores on the DAS. This finding suggests that competition hostility likely evokes professional drivers’ anger in most traffic scenarios. However, the time urgency trait exerts an effect on driving anger only in situations of slow driving and traffic obstructions in both the professional and non-professional driver groups. These results are consistent with earlier findings. Because the increasing levels of TABP in professional drivers increase their degree of driving anger, when selecting professional drivers, those who exhibit high levels of TABP should be chosen prudently. Furthermore, adverse emotions, such as type A behavior tendency, can be examined during driver's license tests to improve the quality of the driver group.

Although the present study used a homogenous sample, namely, professional drivers, this sample is representative of a heterogeneous population. Among professional drivers, TABP translated into driving anger through a fully mediated pathway, and the positive effect of TABPs on anger while driving mediated by the intervening trait anger variable. Therefore, the TABPs exhibited by professional drivers are related to a proclivity to anger, which then spills over into one’s general daily life, such as anger behind the wheel. Thus, levels of trait anger and TABP could be important evaluation indexes for the selection of and training strategies for professional drivers. Furthermore, interventions designed to reduce driving anger and aggression could involve controlling, to a certain degree, TABPs. Additionally, it is unclear whether the translation of type A behavior pattern to anger is a generalizable phenomenon, or whether it is mediated by cultural factors.

### Limitations

This study has some limitations, the greatest of which is the cross-sectional structure of the data because this limits the possibility of drawing decisive conclusions regarding causality. Furthermore, due to a limited the age range among Chinese drivers, which is narrower than that of drivers in Western countries, Chinese drivers have a narrow driving experience range. Thus, this study did not reveal any significant correlations between demographic factors and driving anger. In future work, it is important to enlarge the range of demographic backgrounds when selecting a sample of drivers. In addition, another potential limitation is the sampling bias and the types of professional drivers, which should be broadened to other fields, such as taxi drivers, truckers, etc., to realize greater diversification by capturing more characteristics of professional drivers and thus permit generalization.

Additionally, because the present study is based on drivers’ self-reported data, the results are potentially subjected to the typical limitations associated with a tendency toward social desirability bias. Although the participants were assured of confidentiality and anonymity were geographically separated from each other, the usual weaknesses of questionnaires as research tools cannot be counteracted or completely ruled out.

### Practical implications and directions for further research

While professional and non-professional drivers are two important road users who frequently drive vehicles daily, the differences in the levels of driving anger between these two groups are not receiving adequate attention. Furthermore, whether and the degree to which personality traits spill over into general daily life activities, such as anger behind the wheel, between the two groups of drivers remain in need of further investigation. There are certain exploratory meanings with respect to driver anger that provide a theoretical basis for implementing characteristic and targeted interventions to reduce driver anger and aggressive driving given specific personality traits in different groups of drivers. The findings of this research imply the urgency of work zone management and the necessity of revisiting the enforcement policies regarding road management and construction to reduce traffic obstructions. Additionally, safety can be improved by assisting the corresponding agencies in the development of more effective traffic control and management policies and the enhancement of existing driver education programs. Finally, future work should obtain access to individual driving records because these records may provide objective outcomes to confirm the self-reported information and reduce concerns regarding potential response biases.

## Supporting information

S1 Supporting Information Data for driving anger and its relationships with type A behavior patterns and trait anger. (XLSX)
